# MendelianRandomization v0.5.0: updates to an R package for performing Mendelian randomization analyses using summarized data

**DOI:** 10.12688/wellcomeopenres.16374.2

**Published:** 2020-12-10

**Authors:** Jim R. Broadbent, Christopher N. Foley, Andrew J. Grant, Amy M. Mason, James R. Staley, Stephen Burgess

**Affiliations:** 1 Cardiovascular Epidemiology Unit, University of Cambridge, Cambridge, CB2 0SR, UK; 2 MRC Biostatistics Unit, University of Cambridge, Cambridge, CB1 8RN, UK

**Keywords:** Mendelian randomization, instrumental variable, summarized data, genetic epidemiology, post-GWAS analysis, causal inference, genetic associations.

## Abstract

The MendelianRandomization package is a software package written for the R software environment that implements methods for Mendelian randomization based on summarized data. In this manuscript, we describe functions that have been added to the package or updated in recent years. These features can be divided into four categories: robust methods for Mendelian randomization, methods for multivariable Mendelian randomization, functions for data visualization, and the ability to load data into the package seamlessly from the PhenoScanner web-resource. We provide examples of the graphical output produced by the data visualization commands, as well as syntax for obtaining suitable data and performing a Mendelian randomization analysis in a single line of code.

## Introduction

Mendelian randomization is an epidemiological technique that uses genetic variants to link risk factors to outcomes ^1, 2^. The MendelianRandomization package is a software package written for the R software environment ^3^ that implements methods for Mendelian randomization based on summarized data ^4^. Summarized data are genetic associations with risk factors and outcomes taken from regression analyses that have been performed for each genetic variant in turn ^5^. Such data (beta-coefficients and standard errors) are generated in a genome-wide association study, and have been publicly reported for hundreds of thousands of variants by many large studies and consortia ^6^. While the basic functionality and initial features of the package have been discussed previously ^7^, several functions have been added to the package in recent years. These features can be divided into four categories: robust methods for Mendelian randomization, methods for multivariable Mendelian randomization, functions for data visualization, and the ability to load data into the package seamlessly from the PhenoScanner web-resource. We discuss each of these categories in turn, describing the various options available to investigators. A list of functions in the package is provided as Table 1. We do not discuss in detail the properties of the various methods or the reasons for choosing between the various options presented; we would encourage users to read the relevant references for the methods or the recently-published guidelines paper on performing Mendelian randomization investigations ^8^. We also encourage users to consult the package documentation, which describes all the options available for each method in greater detail. The aim of this paper is to provide a broad overview of the package.

**Table 1.** Functions available in the MendelianRandomization package. Functions are divided into five categories: data entry functions, univariable estimation methods, multivariable estimation methods, data visualization functions, and functions that load data from PhenoScanner.

Function	Description	Status
mr_input mr_mvinput	Data entry for univariable analysis Data entry for multivariable analysis	*
mr_ivw mr_median mr_egger mr_maxlik mr_mbe mr_hetpen mr_conmix mr_lasso mr_allmethods	Inverse-variance weighted (IVW) method Median method MR-Egger method Maximum likelihood method Mode-based estimation method Heterogeneity penalized method Contamination mixture method Lasso method Runs several methods	† * * * *
mr_mvivw mr_mvmedian mr_mvegger mr_mvlasso	Multivariable IVW method Multivariable median-based method Multivariable MR-Egger method Multivariable lasso method	* * * *
mr_plot mr_forest mr_funnel mr_loo	Scatter plot Forest plot Funnel plot Leave-one-out plot	† * * *
extract.pheno.csv pheno_input	Data entry from PhenoScanner .csv ﬁle (legacy) Data entry from web-based PhenoScanner	*

## Methods

### Implementation

The initial release of the MendelianRandomization package included four functions for the estimation of causal effects based on summarized genetic data in a univariable (that is, one risk factor) Mendelian randomization framework. These were mr_ivw (inverse-variance weighted method, IVW) ^9^, mr_egger (MR-Egger method) ^10^, mr_median (simple and weighted median methods) ^11^, and mr_maxlik (maximum likelihood method) ^9^. Each of these estimation functions takes an *MRInput* object as input, created using the mr_input command. The syntax is:


mr_ivw(mr_input(ldlc, ldlcse, chdlodds, chdloddsse))


where ldlc and ldlcse are genetic associations with low-density lipoprotein (LDL) cholesterol and their standard errors for 28 genetic variants as previously reported by Waterworth *et al*. ^12^, and chdlodds and chdloddsse are genetic associations with coronary heart disease risk for the same variants. These data variables are provided with the package. Syntax for the default operation of the mr_egger and mr_median commands (and all the other univariable estimation commands discussed in this paper) is identical, although user-options and the output from each method is different.

Some methods rely on all variants being uncorrelated; others allow correlated variants using the correl option. Using correlated variants requires the specification of the correlation matrix between genetic variants, on the assumption that the correlations between the genetic variants are the same as the correlations between the genetic association estimates ^4^. Correlations are typically estimated from reference data, such as those from European-descent participants of the 1000 Genomes Project that can be obtained using the ld_matrix command in the TwoSampleMR package ^13^. Care should be taken that entries in the correlation matrix are harmonized to the same effect and reference alleles as the genetic associations ^14^; if the correlation matrix was calculated with the effect and reference alleles reversed, then the positive and negative signs should be flipped for the relevant column and row of the matrix (the diagonal terms should remain as +1). Exemplar data on genetic associations with calcium and fasting glucose for correlated variants are provided in the package. The IVW method can be applied to these data using the syntax:.


mr_ivw(mr_input(calcium, calciumse, fastgluc, fastglucse, corr=calc.rho))


where calc.rho is the correlation matrix.

All methods allow confidence intervals to be calculated using a t-distribution rather than a normal distribution ( distribution = "t-dist") or based on a different significance level ( alpha = 0.05 corresponds to a 95% confidence interval). Other options are specific to particular methods; a list of input options for for each method can be found in the package documentation under the subheading “Arguments”; for the mr_ivw method, this is accessed by the command ?mr_ivw.

Each method provides output in a slightly different format. Generally, the estimate from the method is in the *Estimate* slot, its standard error is in the *StdError* slot, and the lower and upper limits of the confidence interval for the estimate are in the *CILower* and *CIUpper* slots. For the mr_ivw command, these can be accessed via:


mr_ivw(mr_input(ldlc, ldlcse, chdlodds, 4chdloddsse))$Estimate
mr_ivw(mr_input(ldlc, ldlcse, chdlodds, chdloddsse))$StdError
mr_ivw(mr_input(ldlc, ldlcse, chdlodds, chdloddsse))$CILower
mr_ivw(mr_input(ldlc, ldlcse, chdlodds, chdloddsse))$CIUpper


A list of output slots for each method can be found in the package documentation under the subheading “Value”; for the mr_ivw method, this is accessed by the command ?mr_ivw.

### Operation

The R software environment runs on a wide variety of UNIX platforms, Windows, and MacOS, and requires minimal computer resources (256 kilobytes of RAM is recommended). The package requires R version 3.0.1 or higher.

## Use cases

### Robust methods for Mendelian randomization

A brief description of each method is given in Table 2. These methods were discussed in greater detail and compared in a review of robust methods for Mendelian randomization ^15^.

**Table 2.** Comparison of univariable methods implemented in the MendelianRandomization package. A more detailed comparison of robust methods for Mendelian randomization can be found in a recent review ^15^. Abbreviation: InSIDE = instrument strength independent of direct effect.

Method	Function name	Strengths and weaknesses	Reference
Inverse variance weighted MR-Egger Median Maximum likelihood	mr_ivw mr_egger mr_median mr_maxlik	Most efficient (greatest statistical power), biased if average pleiotropic effect differs from zero Robust to pleiotropy under InSIDE assumption, sensitive to outliers, sensitive to violations of InSIDE assumption, InSIDE assumption often not plausible, often less efficient Robust to outliers, sensitive to addition/removal of genetic variants Similar to IVW method, accounts for uncertainty in genetic associations with risk factor	9 10 11 9
MR-Robust Penalized weights Mode-based estimation Heterogeneity- penalized MR-Lasso Contamination mixture	mr_ivw(…, robust=TRUE) mr_ivw(…, penalized=TRUE) mr_mbe mr_hetpen mr_lasso mr_conmix	Downweights outliers, efficient with valid IVs, high false positive rate with several invalid IVs Downweights outliers, efficient with valid IVs, high false positive rate with several invalid IVs Robust to outliers, sensitive to bandwidth parameter and addition/removal of genetic variants, often less efficient Robust to outliers, can only be implemented for a small number of variants due to computational efficiency Removes outliers, efficient with valid IVs, high false positive rate with several invalid IVs Robust to outliers, sensitive to variance parameter and addition/removal of genetic variants	16 16 17 18 16 19

The IVW method is implemented by weighted linear regression of the genetic associations with the outcome on the genetic associations with the risk factor ^4^. There are two options in the mr_ivw method that represent different robust methods. The robust option performs the IVW method method using robust regression (referred to as MR-Robust) ^16^. The penalized option performs the IVW method with penalized weights ^16^. The syntax is:


mr_ivw(mr_input(ldlc, ldlcse, chdlodds, chdloddsse), robust=TRUE)
mr_ivw(mr_input(ldlc, ldlcse, chdlodds, chdloddsse), penalized=TRUE)


Other methods implemented in the package are the mode-based method ( mr_mbe) ^17^, the heterogeneity penalized method ( mr_hetpen) ^18^, the lasso method ( mr_lasso) ^16^, and the contamination mixture method ( mr_conmix) ^19^. As for the mr_ivw command, the syntax is:


mr_mbe(mr_input(ldlc, ldlcse, chdlodds, chdloddsse))


and similarly for the other methods.

The mr_mbe method has options weighting = "weighted" or weighting = "simple", corresponding to weighted and unweighted versions of the method. It also has options stderror = "simple" or stderror = "delta" corresponding to first- and second-order standard errors. 

The mr_hetpen method has options prior to set the prior probability of a genetic variant being a valid instrument (default is 0.5), and CIMin, CIMax, and CIStep to allow feasible and efficient calculation of confidence intervals.

The mr_conmix method has options psi to set the value of the standard deviation of the distribution of invalid estimands (that is, how variable are the quantities targeted by genetic variants that are invalid instrumental variables), and CIMin, CIMax, and CIStep as above. 

The mr_lasso method has the option lambda to set the tuning parameter in the penalized (lasso) regression model.

### Methods for multivariable Mendelian randomization

Multivariable Mendelian randomization is an extension of the standard Mendelian randomization paradigm to include multiple risk factors in a single analysis model ^20, 21^. Typically, it is employed when several risk factors share genetic predictors, and so it is not possible to find genetic variants that are specific predictors of a particular risk factor. In multivariable Mendelian randomization, it is assumed that the genetic variants are specifically associated with any of a set of risk factors, such that any causal pathway from the genetic variants to the outcome passes via one or other of the risk factors. To perform multivariable Mendelian randomization with summarized data, genetic associations are required for each variant with all of the risk factors.

Methods for multivariable Mendelian randomization take an *MRMVInput* object as an input, created using the mr_mvinput command. Four functions are included for the estimation of causal effects based on summarized genetic data in a multivariable Mendelian randomization framework. These are mr_mvivw (multivariable IVW method) ^22^, mr_mvegger (multivariable MR-Egger method) ^23^, mr_mvmedian (multivariable median-based method) ^24^, and mr_mvlasso (multivariable lasso method). The syntax is:


mr_mvivw(mr_mvinput(bx = cbind(ldlc, hdlc, trig),
   bxse = cbind(ldlcse, hdlcse, trigse),
   by = chdlodds, byse = chdloddsse))


where hdlc and hdlcse are genetic associations with high-density lipoprotein (HDL) cholesterol and their standard errors, and trig and trigse are genetic associations with triglycerides and their standard errors for the same 28 variants. Again, these data variables are provided with the package. Syntax for the mr_mvegger, mr_mvmedian, and mr_mvlasso commands is identical. 

The multivariable IVW method is implemented similarly to the univariable IVW method, except using multivariable regression of the genetic associations with the outcome on the genetic associations with the risk factors. As in the univariable case, the mr_mvivw command can be implemented using robust regression with the robust = TRUE option ^24^. The mr_mvivw and mr_mvegger methods have a correl option to allow for correlated variants. The mr_mvlasso method has the lambda option to set the penalization parameter as in the univariable case. All methods have distribution and alpha options as discussed above.

### Functions for data visualization

The initial release of the MendelianRandomization package included two options for data visualization, both implemented using the mr_plot function. Application of the mr_plot function to an *MRInput* object gave an interactive scatter plot of the genetic associations together with a line representing the IVW estimate. Genetic associations with the risk factor are plotted on the horizontal axis, and genetic associations with the outcome on the vertical axis. Application of the mr_plot function to an *MRAll* object plotted a similar (although non-interactive) scatter plot of the genetic associations together with lines representing the estimates from various methods. An *MRAll* object is created by the mr_allmethods function, which returns estimates from various estimation methods.

**Figure 1.**
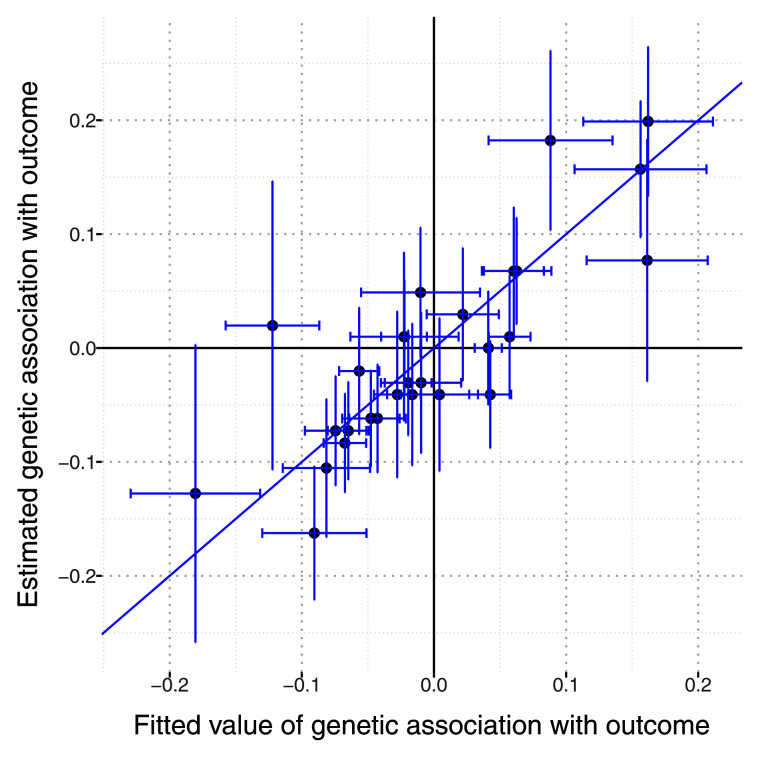
Scatter plot created by mr_plot command applied to a *MRMVInput* object. Estimated genetic associations with the outcome (vertical axis) are plotted against predicted associations with the outcome from the multivariable inverse-variance weighted method (horizontal axis). Error bars are 95% confidence intervals, and the diagonal line has gradient 1.

We have added functionality so that the mr_plot function can now be applied to an *MRMVInput* object. In this case, we still plot the estimated genetic associations with the outcome on the vertical axis. On the horizontal axis, we plot predicted genetic associations with the outcome. These are fitted values from the multivariable IVW method, which regresses the genetic associations with the outcome on the genetic associations with the risk factors. Horizontal error bars represent confidence intervals for these fitted values. These refiect uncertainty in the multivariable IVW estimates, but not in the genetic associations with the risk factors, which are assumed to be known without error. A diagonal line is plotted with gradient 1 to help the detection of outliers, which may be pleiotropic variants. The syntax is:


mr_plot(mr_mvinput(bx = cbind(ldlc, hdlc, trig), # Figure 1
                   bxse = cbind(ldlcse, hdlcse, trigse),
                   by = chdlodds, byse = chdloddsse))


In the example of Figure 1, we additionally set the option interactive = FALSE to produce a non-interactive version of this plot.

**Figure 2.**
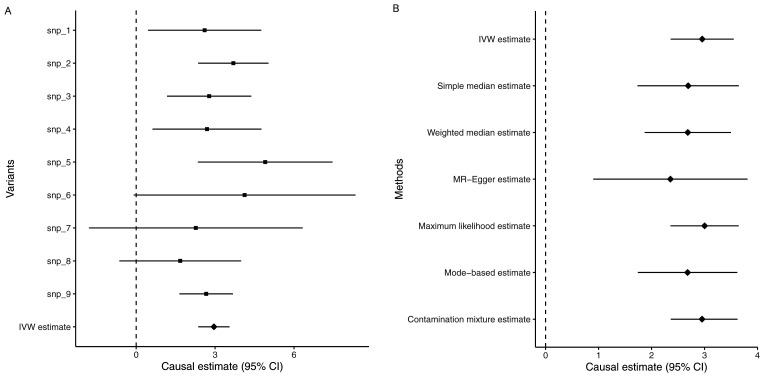
Forest plots created by mr_forest command. Left panel: comparison of variant-specific estimates plus inverse-variance weighted (IVW) estimate (default options). Right panel: comparison of estimates from different methods with variant-specific estimates switched off. Points represent estimates and horizontal error bars are 95% confidence intervals (CI).

**Figure 3.**
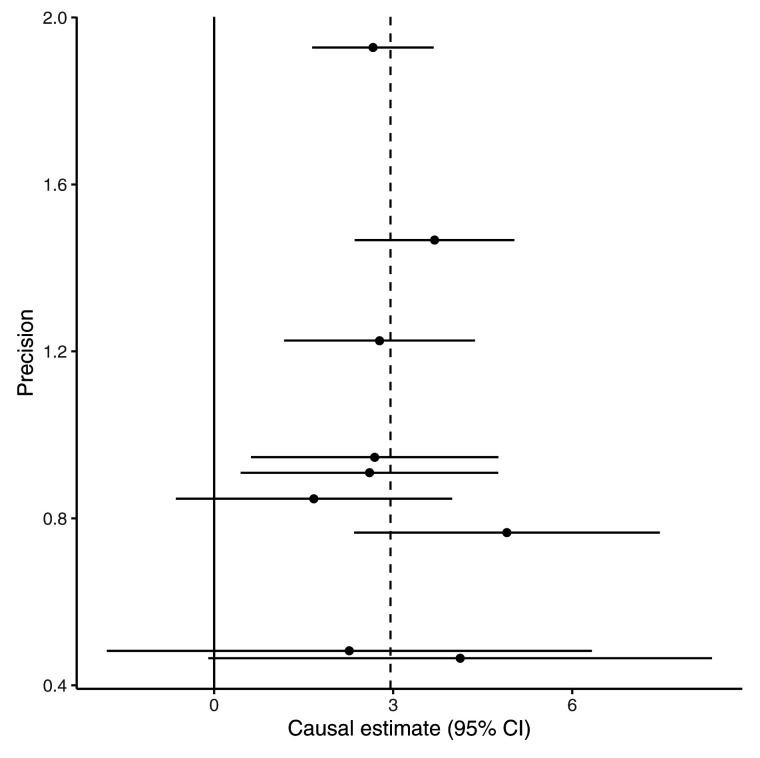
Funnel plot created by mr_funnel command. Points represent variant-specific estimates and horizontal error bars are 95% confidence intervals (CI).

In updating the package, we have added several additional functions for data visualization. The default implementation of the mr_forest function plots the variant-specific estimates in a forest plot, with the pooled estimate from the IVW method at the bottom ( Figure 2a). The variant-specific estimates are the ratio estimates from each genetic variant in turn. This plot allows the user to investigate heterogeneity in the variant-specific estimates, which indicates potential pleiotropy in the analysis ^25^. Heterogeneity can also be expressed numerically by Cochran's Q statistic (for the IVW method) or Rücker's Q statistic (for the MR-Egger method), which are reported as the “heterogeneity test statistic” by the relevant estimation functions. The mr_forest function can also be used to plot estimates from different methods, either in addition to the variant-specific estimates or without them ( Figure 2b):


mr_forest(mr_input(ldlc, ldlcse, chdlodds, chdloddsse)) # Figure 2A
mr_forest(mr_input(ldlc, ldlcse, chdlodds, chdloddsse), # Figure 2B
    snp_estimates=FALSE,
    methods = c("ivw", "median", "wmedian", "egger", "maxlik", "mbe", "conmix"))


(For presentation purposes, in this and subsequent figures we provide plots for the first 9 variants in the package only.) The mr_funnel function is similar, except that the variant-specific estimates are plotted against their precision (that is, the reciprocal of their standard error). This plot also enables the user to investigate heterogeneity in the variant-specific estimates ( Figure 3):


mr_funnel(mr_input(ldlc, ldlcse, chdlodds, chdloddsse)) # Figure 3


**Figure 4.**
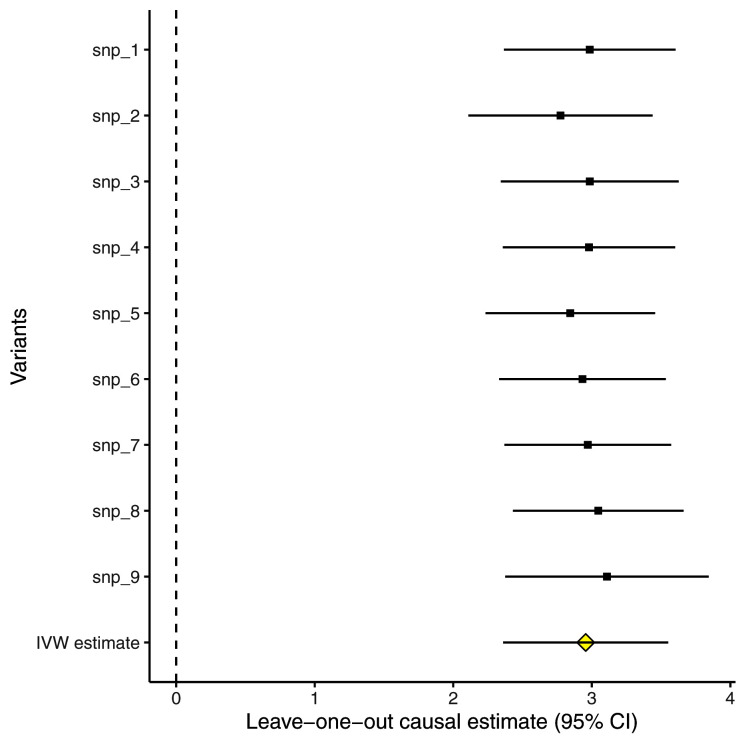
Leave-one-out plot created by mr_loo command. Points represent estimates from the inverse-variance weighted (IVW) method, omitting the variant indicated. Horizontal error bars are 95% confidence intervals (CI).

The mr_loo function allows the user to investigate sensitivity of the IVW estimate to individual data points. This is implemented by calculating the IVW estimate omitting each variant from the analysis in turn (loo stands for ‘leave one out’). The IVW estimate based on all the variants is also plotted for reference ( Figure 4):


mr_loo(mr_input(ldlc, ldlcse, chdlodds, chdloddsse)) # Figure 4


Output from each of these commands is a *ggplot* object, and so basic graphical parameters can be changed using functions from the ggplot2 package ^26^. For example, the horizontal axis can be set to run from −5 to +5 using the following code:


library(ggplot2)
forest  = mr_forest(mr_input(ldlc, ldlcse, chdlodds, chdloddsse))
forest2 = forest + coord_cartesian(xlim=c(-5,5))
forest2


### Loading data from PhenoScanner

The initial release of the MendelianRandomization package included a function called extract.pheno.csv. This function took a .csv file previously downloaded by the user from the PhenoScanner webtool ( http://www.phenoscanner.medschl.cam.ac.uk/) and converted the file into an *MRInput* object, extracting the relevant genetic associations with the risk factor and outcome. PhenoScanner ^27, 28^ is a database of genetic associations that contains over 65 billion associations for over 150 million unique genetic variants, including genetic associations reported by major consortia, as well as those for the UK Biobank study reported by Ben Neale’s team ( http://www.nealelab.is/uk-biobank). 

The extract.pheno.csv function is no longer maintained; however, it has been superseded by the pheno_input command, which calls PhenoScanner directly from R and creates an *MRInput* object. Using this command, the entire workflow of a Mendelian randomization analysis can be performed in a single line of code. For example:


mr_ivw(pheno_input(snps=c("rs12916", "rs2479409", "rs217434", "rs1367117",
                          "rs4299376", "rs629301", "rs4420638", "rs6511720"),
 exposure = "Low density lipoprotein", pmidE = "24097068", ancestryE = "European",
 outcome = "Coronary artery disease", pmidO = "26343387", ancestryO = "Mixed"))


This code first extracts data on eight genetic variants (their ‘rsid’ identifiers are listed above), and creates an *MRInput* object using the genetic associations with “low density lipoprotein” taken from the study with PubMed ID 24097068 ^29^ in individuals of European descent as the summarized associations with the risk factor, and genetic associations with “coronary artery disease” taken from the study with PubMed ID 26343387 ^30^ in a mixed ancestry sample as the summarized associations with the outcome. The triplet of trait name, PubMed ID, and ancestry is necessary to uniquely identify the correct dataset for genetic associations, as some publications report associations with multiple traits, or associations with the same trait in different ancestry groups. While the above code then implements the IVW method on this *MRInput* object, any other estimation or data visualization command that takes an *MRInput* object as input could be applied to the output of the pheno_input function.

## Summary

In summary, the MendelianRandomization package has added a number of features since its initial release: to implement various robust estimation methods, to implement methods for multivariable Mendelian randomization, to enable a greater range of data visualization options, and to facilitate data entry. We conclude with the same warning that we stated at the end of the manuscript accompanying the initial package release ^7^: while this software simplifies the operational aspects of a Mendelian randomization, the truly difficult parts of an analysis are choosing sensible risk factors and outcomes, selecting genetic variants that are plausible instrumental variables, performing a reasonable range of analyses, and interpreting the results with care and caution ^31^. Software code for these aspects of an analysis cannot be written ^32^.

## Data availability

### Underlying data

All data used in this article are distributed in the software package described, or can be freely downloaded using commands in the software package that are detailed in the text of the article.

## Software availability

The MendelianRandomization package is available via the Comprehensive R Archive Network (CRAN)

The software package is available here: https://cran.r-project.org/web/packages/MendelianRandomization/index.html. 

Source code is available from GitHub: https://github.com/sb452/MendelianRandomization/tree/v0.5.0

Archived source code at time of publication: http://doi.org/10.5281/zenodo.4088672 ^33^

Software license: GPL-2 | GPL-3. 

## References

[1] Smith GD Ebrahim S: 'Mendelian randomization': can genetic epidemiology contribute to understanding environmental determinants of disease? * Int J Epidemiol.* 2003;32(1):1–22. 10.1093/ije/dyg070 12689998

[2] Burgess S Thompson SG: Mendelian randomization: methods for using genetic variants in causal estimation. Chapman & Hall, Boca Raton, FL.2015 10.1201/b18084

[3] R Core Team: R: A language and environment for statistical computing. Version 4.0.2 (Taking Off Again). R Foundation for Statistical Computing, Vienna, Austria,2020 Reference Source

[4] Burgess S Dudbridge F Thompson SG: Combining information on multiple instrumental variables in Mendelian randomization: comparison of allele score and summarized data methods. * Stat Med.* 2016;35(11):1880–1906. 10.1002/sim.6835 26661904 PMC4832315

[5] Bowden J Del Greco FM Minelli C: A framework for the investigation of pleiotropy in two-sample summary data Mendelian randomization. *Stat Med.* 2017;36(11):1783–1802. 10.1002/sim.7221 28114746 PMC5434863

[6] Burgess S Scott RA Timpson NJ: Using published data in Mendelian randomization: a blueprint for efficient identification of causal risk factors. *Eur J Epidemiol.* 2015;30(7):543–552. 10.1007/s10654-015-0011-z 25773750 PMC4516908

[7] Yavorska OO Burgess S: MendelianRandomization: an R package for performing Mendelian randomization analyses using summarized data. *Int J Epidemiol.* 2017;46(6):1734–1739. 10.1093/ije/dyx034 28398548 PMC5510723

[8] Burgess S Smith GD Davies NM: Guidelines for performing Mendelian randomization investigations [version 2; peer review: 2 approved]. *Wellcome Open Res.* 2020;4:186. 10.12688/wellcomeopenres.15555.2 32760811 PMC7384151

[9] Burgess S Butterworth A Thompson SG: Mendelian randomization analysis with multiple genetic variants using summarized data. *Genet Epidemiol.* 2013;37(7):658–665. 10.1002/gepi.21758 24114802 PMC4377079

[10] Bowden J Smith GD Burgess S: Mendelian randomization with invalid instruments: effect estimation and bias detection through Egger regression. *Int J Epidemiol.* 2015;44(2):512–525. 10.1093/ije/dyv080 26050253 PMC4469799

[11] Bowden J Smith GD Haycock PC: Consistent Estimation in Mendelian Randomization with Some Invalid Instruments Using a Weighted Median Estimator. *Genet Epidemiol.* 2016;40(4):304–314. 10.1002/gepi.21965 27061298 PMC4849733

[12] Waterworth DM Ricketts SL Song K: Genetic variants influencing circulating lipid levels and risk of coronary artery disease. *Arterioscler Thromb Vasc Biol.* 2010;30(11):2264–2276. 10.1161/ATVBAHA.109.201020 20864672 PMC3891568

[13] Hemani G Zheng J Elsworth B: The MR-Base platform supports systematic causal inference across the human phenome. *eLife.* 2018;7:e34408. 10.7554/eLife.34408 29846171 PMC5976434

[14] Hartwig FP Davies NM Hemani G: Two-sample Mendelian randomization: avoiding the downsides of a powerful, widely applicable but potentially fallible technique. *Int J Epidemiol.* 2016;45(6):1717–1726. 10.1093/ije/dyx028 28338968 PMC5722032

[15] Slob EAW Burgess S: A comparison of robust Mendelian randomization methods using summary data. *Genet Epidemiol.* 2020;44(4):313–329. 10.1002/gepi.22295 32249995 PMC7317850

[16] Rees JMB Wood AM Dudbridge F: Robust methods in Mendelian randomization via penalization of heterogeneous causal estimates. *PLoS One.* 2019;14(9):e0222362. 10.1371/journal.pone.0222362 31545794 PMC6756542

[17] Hartwig FP Smith GD Bowden J: Robust inference in summary data Mendelian randomization via the zero modal pleiotropy assumption. *Int J Epidemiol.* 2017;46(6):1985–1998. 10.1093/ije/dyx102 29040600 PMC5837715

[18] Burgess S Zuber V Gkatzionis A: Modal-based estimation via heterogeneity-penalized weighting: model averaging for consistent and efficient estimation in Mendelian randomization when a plurality of candidate instruments are valid. * Int J Epidemiol.* 2018;47(4):1242–1254. 10.1093/ije/dyy080 29846613 PMC6124628

[19] Burgess S Foley CN Allara E: A robust and efficient method for Mendelian randomization with hundreds of genetic variants. * Nat Commun.* 2020;11:376. 10.1038/s41467-019-14156-4 31953392 PMC6969055

[20] Burgess S Thompson SG: Multivariable Mendelian randomization: the use of pleiotropic genetic variants to estimate causal effects. *Am J Epidemiol.* 2015;181(4):251–260. 10.1093/aje/kwu283 25632051 PMC4325677

[21] Sanderson E Davey Smith G Windmeijer F: An examination of multivariable Mendelian randomization in the single-sample and two-sample summary data settings. * Int J Epidemiol.* 2019;48(3):713–727. 10.1093/ije/dyy262 30535378 PMC6734942

[22] Burgess S Dudbridge F Thompson SG: Re: “Multivariable Mendelian randomization: the use of pleiotropic genetic variants to estimate causal effects”. *Am J Epidemiol.* 2015;181(4):290–291. 10.1093/aje/kwv017 25660081

[23] Rees JMB Wood AM Burgess S: Extending the MR-Egger method for multivariable Mendelian randomization to correct for both measured and unmeasured pleiotropy. *Stat Med.* 2017;36(29):4705–4718. 10.1002/sim.7492 28960498 PMC5725762

[24] Grant AJ Burgess S: Pleiotropy robust methods for multivariable Mendelian randomization. *arXiv.2008.11997.* 2020 Reference Source 10.1002/sim.9156 PMC7612169 34342032

[25] Bowden J Hemani G Davey Smith G: Detecting individual and global horizontal pleiotropy in Mendelian randomization – a job for the humble heterogeneity statistic? *Am J Epidemiol.* 2018;187(12):2681–2685. 10.1093/aje/kwy185 30188969 PMC6269239

[26] Wickham H: ggplot2: Elegant Graphics for Data Analysis.Springer-Verlag New York,2016 Reference Source

[27] Staley JR Blackshaw J Kamat MA: PhenoScanner: a database of human genotype-phenotype associations. *Bioinformatics.* 2016;32(20):3207–3209. 10.1093/bioinformatics/btw373 27318201 PMC5048068

[28] Kamat MA Blackshaw JA Young R: PhenoScanner V2: an expanded tool for searching human genotype–phenotype associations. *Bioinformatics.* 2019;35(22):4851–4853. 10.1093/bioinformatics/btz469 31233103 PMC6853652

[29] Willer CJ Schmidt EM Sengupta S: Discovery and refinement of loci associated with lipid levels. *Nat Genet.* 2013;45(11):1274–1283. 10.1038/ng.2797 24097068 PMC3838666

[30] Nikpay M Goel A Won HH: A comprehensive 1000 Genomes-based genome-wide association meta-analysis of coronary artery disease. * Nat Genet.* 2015;47(10):1121–1130. 10.1038/ng.3396 26343387 PMC4589895

[31] Burgess S Davey Smith G Davies NM: Guidelines for performing Mendelian randomization investigations [version 2; peer review: 2 approved]. *Wellcome Open Res.* 2020;4:186. 10.12688/wellcomeopenres.15555.2 32760811 PMC7384151

[32] Burgess S Davey Smith G: How humans can contribute to Mendelian randomization analyses. *Int J Epidemiol.* 2019;48(3):661–664. 10.1093/ije/dyz152 31326987 PMC6739231

[33] sb452: sb452/mendelianrandomization: Mendelianrandomization version 0.5.0.2020 10.5281/zenodo.4088672

