# Supplementary material for: MendelianRandomization v0.5.0: updates to an R package for performing Mendelian randomization analyses using summarized data
Source: Wellcome Open Res. 2020 Dec 10;5:252. Originally published 2020 Oct 22. [Version 2] doi: 10.12688/wellcomeopenres.16374.2 (PMC7745186; doi:10.12688/wellcomeopenres.16374.2)
Supplement: Peer review history

# Peer review: Reviewer response for version 2

**DOI:** 10.21956/wellcomeopenres.18125.r41795

**Published:** 2020-12-16

**Authors:** Marilyn C. Cornelis

**Affiliations:** 1 Department of Preventive Medicine, Feinberg School of Medicine, Northwestern University, Chicago, IL, USA

The authors have addressed my concerns.

Are the conclusions about the tool and its performance adequately supported by the findings presented in the article?

Yes

Is the rationale for developing the new software tool clearly explained?

Yes

Is the description of the software tool technically sound?

Yes

Are sufficient details of the code, methods and analysis (if applicable) provided to allow replication of the software development and its use by others?

Yes

Is sufficient information provided to allow interpretation of the expected output datasets and any results generated using the tool?

Yes

Reviewer Expertise:

genetic epidemiology, nutrition, cardiometabolic traits, aging traits, coffee, caffeine, metabolomics

I confirm that I have read this submission and believe that I have an appropriate level of expertise to confirm that it is of an acceptable scientific standard.

# Peer review: Reviewer response for version 1

**DOI:** 10.21956/wellcomeopenres.18010.r41072

**Published:** 2020-11-06

**Authors:** Marilyn C. Cornelis

**Affiliations:** 1 Department of Preventive Medicine, Feinberg School of Medicine, Northwestern University, Chicago, IL, USA

Broadbent *et al.* summarize updates made to their MendelianRandomization R package. The package is widely used and thus a detailed description of updates is justified and warranted. I have only very minor suggestions.

1. The R package manual (updated September 30, 2020) details all functions described in this paper. The introduction or summary should briefly mention what the current paper adds above and beyond the R package manual.
2. The paper can use some minor re-organizing or heading revisions. Currently, “Introduction”, “Methods”, “Use cases”, “Summary”, “Data availability” and “Software availability” are the major sections. The “Use cases” is well written and describes features in four categories which the authors introduce in the beginning and constitutes the key/main section of the paper. The “Methods” section, however, seems out of place or mislabeled for this descriptive paper. Perhaps use “Software overview”?
3. Table 1. Please annotate functions that are new and not discussed in reference 5.

Are the conclusions about the tool and its performance adequately supported by the findings presented in the article?

Yes

Is the rationale for developing the new software tool clearly explained?

Yes

Is the description of the software tool technically sound?

Yes

Are sufficient details of the code, methods and analysis (if applicable) provided to allow replication of the software development and its use by others?

Yes

Is sufficient information provided to allow interpretation of the expected output datasets and any results generated using the tool?

Yes

Reviewer Expertise:

genetic epidemiology, nutrition, cardiometabolic traits, aging traits, coffee, caffeine, metabolomics

I confirm that I have read this submission and believe that I have an appropriate level of expertise to confirm that it is of an acceptable scientific standard, however I have significant reservations, as outlined above.

# Response

**Published:** 2020-11-20

**Authors:** Stephen Burgess

**Affiliations:** University of Cambridge Addenbrooke's Hospital Cambridge, UK

**Reviewer 3: Marilyn C. Cornelis**

*C0. Broadbent et al. summarize updates made to their MendelianRandomization R package. The package is widely used and thus a detailed description of updates is justified and warranted. I have only very minor suggestions.*

 - We thank the reviewer for their positive view of this submission. 

*C1.    The R package manual (updated September 30, 2020) details all functions described in this paper. The introduction or summary should briefly mention what the current paper adds above and beyond the R package manual.*

 - The reviewer is correct that all of the material in the current paper is also available through the R package manual. However, there are reasons why we believe that a publication is worthwhile. First, this paper presents the contents of the package in a clearer and more holistic way than the manual. Secondly, it provides examples of usage and interpretation of the output. Thirdly, it creates a citation in the scientific literature so that we can track usage of the package.

 - We have added text to the introduction to more clearly define the scope of this paper. We have also referenced the manual more clearly within the text, for example to obtain lists of the input options and output slots for each method. 

*C2.    The paper can use some minor re-organizing or heading revisions. Currently, “Introduction”, “Methods”, “Use cases”, “Summary”, “Data availability” and “Software availability” are the major sections. The “Use cases” is well written and describes features in four categories which the authors introduce in the beginning and constitutes the key/main section of the paper. The “Methods” section, however, seems out of place or mislabeled for this descriptive paper. Perhaps use “Software overview”?*

 - Thank you for the suggestion. However, the format of the article is set by the journal to ensure homogeneity of papers across this article class. Hence we do not have complete freedom to determine the structure of the article. We have reviewed the guidelines and re-arranged where possible according to the journal guidelines, but the original structure largely remains. 

*C3.    Table 1. Please annotate functions that are new and not discussed in reference 5.*

 - We have added annotation of those functions that are new and those that are updated since the publication of Yavorska and Burgess 2017 (now reference 7).

# Peer review: Reviewer response for version 1

**DOI:** 10.21956/wellcomeopenres.18010.r41070

**Published:** 2020-11-05

**Authors:** Wes Spiller

**Affiliations:** 1 Department of Population Health Sciences, University of Bristol, Bristol, UK

This article demonstrates how a range of valuable Mendelian randomization approaches can be easily implemented using the MendelianRandomization R Package. It describes substantial additions to the existing software, which I believe would be of great value to researchers. This is especially true of integrating data from the PhenoScanner web-resource, which can be invaluable in identifying avenues for pleiotropic bias.

 The material presented in the article is technically sound, though I believe it could be improved with minor revisions.

1. In the methods section, some of the wording is at times confusing, for example: “simple and weighted method methods)”.
2. There is an understandably difficult balance between summarising methods previously described, and providing sufficient detail so as for researchers to understand how options relate to methods implemented. For example, while the correl option is referenced several times, there is no indication of appropriate usage (such as providing a suitable correlation matrix). Especially in the *Use cases* section, the details provided at times read like the help file for various functions when using R. This shifts from providing insufficient detail overall for using such methods (specifically novel options provided in the package) with confidence, to providing at times a puzzling focus on elements such as the standard error options for the modal estimator. If these options are important enough to be included in the manuscript, then this would warrant more description of their usage.
3. A brief overview of multivariable MR would potentially be helpful, as there is often confusion over whether only SNPs correlated with all exposures should be used, or a set of SNPs where each SNP is correlated with at least one exposure included in the model. I appreciate this has been explained in previous publications, but the clarification would be welcome.

Having performed an example analysis using this software as part of the review process, I commend the authors on providing helpful vignettes and documentation guiding the analysis process in the R package itself.

 It is also very encouraging to see issues surrounding the appropriate selection of exposures and outcomes, as well as interpretation of results, afforded attention at times lacking in software showcasing novel statistical approaches.

 With the above comments in mind, I believe the MendelianRandomization R package represents a valuable addition to a growing body of MR software ensuring emerging methods are easily implemented in applied research.

Are the conclusions about the tool and its performance adequately supported by the findings presented in the article?

Yes

Is the rationale for developing the new software tool clearly explained?

Yes

Is the description of the software tool technically sound?

Yes

Are sufficient details of the code, methods and analysis (if applicable) provided to allow replication of the software development and its use by others?

Partly

Is sufficient information provided to allow interpretation of the expected output datasets and any results generated using the tool?

No

Reviewer Expertise:

Genetic Epidemiology, Applied Statistics, Mendelian Randomization, Transethnic MR

I confirm that I have read this submission and believe that I have an appropriate level of expertise to confirm that it is of an acceptable scientific standard, however I have significant reservations, as outlined above.

# Response

**Published:** 2020-11-20

**Authors:** Stephen Burgess

**Affiliations:** University of Cambridge Addenbrooke's Hospital Cambridge, UK

**Reviewer 2: Wes Spiller**

*B0. This article demonstrates how a range of valuable Mendelian randomization approaches can be easily implemented using the MendelianRandomization R Package. It describes substantial additions to the existing software, which I believe would be of great value to researchers. This is especially true of integrating data from the PhenoScanner web-resource, which can be invaluable in identifying avenues for pleiotropic bias.*

 - We thank the reviewer for their positive view of this submission. 

*The material presented in the article is technically sound, though I believe it could be improved with minor revisions.*

*B1.    In the methods section, some of the wording is at times confusing, for example: “simple and weighted method methods)”.*

 - Thank you for pointing out that error. The text should read “simple and weighted median methods”. 

*B2.    There is an understandably difficult balance between summarising methods previously described, and providing sufficient detail so as for researchers to understand how options relate to methods implemented. For example, while the correl option is referenced several times, there is no indication of appropriate usage (such as providing a suitable correlation matrix). Especially in the Use cases section, the details provided at times read like the help file for various functions when using R. This shifts from providing insufficient detail overall for using such methods (specifically novel options provided in the package) with confidence, to providing at times a puzzling focus on elements such as the standard error options for the modal estimator. If these options are important enough to be included in the manuscript, then this would warrant more description of their usage.*

 - We appreciate the sentiment of the reviewer’s comment. As per the response to A3, our preference is for this paper to be brief and to illustrate the options available rather than to wade into deep discussions about the properties of the various methods or the reasoning for choosing between the different options available. Hence, we are happy to provide the various options that can be chosen, but we would rather not discuss the merits and demerits of the options at length – these are already addressed elsewhere. This is now explicitly stated in the Introduction.

 - If there are other specific examples where the reviewer feels that additional detail is required, then we would be happy to consider adding these. As for the specific examples that the reviewer calls out: 1) we are not aware of any novel options that are provided in the package – all the methods and options have been introduced elsewhere (see citations in the new Table 2); 2) we have added explanation and an example of the “correl” option; 3) the discussion of standard errors for the mode-based estimation method is one sentence. We do not think this is excessive. The original version of the mode-based estimation method provided by the authors gave 12 different estimates based on different options for the method (3 options for the phi parameter, weighted and unweighted, simple and second-order standard errors): we have condensed these down into the three options discussed in the submission. 

*B3.    A brief overview of multivariable MR would potentially be helpful, as there is often confusion over whether only SNPs correlated with all exposures should be used, or a set of SNPs where each SNP is correlated with at least one exposure included in the model. I appreciate this has been explained in previous publications, but the clarification would be welcome.*

 - As per the response to A4, we have added a short description of multivariable MR to the manuscript. In particular, we clarify that genetic associations are required for all variants with each risk factor. 

*B4. Having performed an example analysis using this software as part of the review process, I commend the authors on providing helpful vignettes and documentation guiding the analysis process in the R package itself.*

 - We thank the reviewer for his positive feedback on the vignettes and documentation. 

*B5. It is also very encouraging to see issues surrounding the appropriate selection of exposures and outcomes, as well as interpretation of results, afforded attention at times lacking in software showcasing novel statistical approaches.*

 - We thank the reviewer for his positive feedback on our discussion of practical implementation of the methods and interpretation of the results. 

*B6. With the above comments in mind, I believe the MendelianRandomization R package represents a valuable addition to a growing body of MR software ensuring emerging methods are easily implemented in applied research.*

 - We thank the reviewer for his comments, which have strengthened and clarified this manuscript.

# Peer review: Reviewer response for version 1

**DOI:** 10.21956/wellcomeopenres.18010.r41160

**Published:** 2020-11-02

**Authors:** Karl Smith Byrne

**Affiliations:** 1 The International Agency for Research on Cancer, Lyon, France

This article describes an invaluable addition to the set of tools facilitating MR analyses, and provides a more up-to-date suite of methods.  

 I believe the paper, however, would benefit from some minor revisions:

1. In the introduction I advise the authors to amend the specification that MR is used on ‘modifiable’ risk factors and ‘clinical’ outcomes as MR can and often is conducted for risk factors that are not clearly modifiable, such as proteins, and similarly MR may be run on non-clinical outcomes.
2. Additionally, I would provide greater clarity for ‘summarised data’; perhaps, summary level data for the association of SNPs with a trait, or facilitate a two-sample MR design. 
3. For the description of methods, I would firstly comment that the degree of customisability of these methods (mr_mbe, for example), is rather impressive and to be lauded. However, I would perhaps add one or two sentences to each of the method paragraphs further clarifying the use of these options. For example, the description of the priors of SNPs with causal effects may be quite unclear for those coming new to these methods. While I acknowledge that ultimately the papers behind each method should be read by users of the software, one or two extra helper sentences for each method would be an easy way to improve the ease of use for your software.
4. Similarly, it would be useful to add some extra description to the MVMR introduction to explicitly specify what is going on; for example that the basic MVMR is just a regression with first order weights (y ~ x1 +x2) to make it less intimidating for users.
5. Is there a facility to report Cochran’s or ruckers Q within the software? The authors discuss the value of visually inspecting plots for heterogeneity but it would wonderful if it were possible to return the statistics for this. 
6. I also note that it is enjoyable that the plots can be directly edited as ggplot objects.
7. Finally, it would be excellent to have a brief paragraph explaining the format of the data frame of results being reported. It can be quite frustrating when using a package to have to manually explore the data object to try and sleuth out what exactly is present and what it means. I expect this is in the vignette in R but I recommend a summary of this here, perhaps, even only for the components reported in common for all MR methods.

In general, this is a welcome update that is described in a clear manner. With the minor updates I suggest above it will be a very useful tool for genetic epidemiology.

Are the conclusions about the tool and its performance adequately supported by the findings presented in the article?

Yes

Is the rationale for developing the new software tool clearly explained?

Yes

Is the description of the software tool technically sound?

Yes

Are sufficient details of the code, methods and analysis (if applicable) provided to allow replication of the software development and its use by others?

Partly

Is sufficient information provided to allow interpretation of the expected output datasets and any results generated using the tool?

No

Reviewer Expertise:

Genetic and Molecular Cancer Epidemiology

I confirm that I have read this submission and believe that I have an appropriate level of expertise to confirm that it is of an acceptable scientific standard, however I have significant reservations, as outlined above.

# Response

**Published:** 2020-11-20

**Authors:** Stephen Burgess

**Affiliations:** University of Cambridge Addenbrooke's Hospital Cambridge, UK

**Reviewer 1: Karl Smith Byrne**

*A0. This article describes an invaluable addition to the set of tools facilitating MR analyses, and provides a more up-to-date suite of methods.*

 - We thank the reviewer for their positive view of this submission. 

*I believe the paper, however, would benefit from some minor revisions:*

*A1.    In the introduction I advise the authors to amend the specification that MR is used on ‘modifiable’ risk factors and ‘clinical’ outcomes as MR can and often is conducted for risk factors that are not clearly modifiable, such as proteins, and similarly MR may be run on non-clinical outcomes.*

 - We have modified the language as suggested. 

*A2.    Additionally, I would provide greater clarity for ‘summarised data’; perhaps, summary level data for the association of SNPs with a trait, or facilitate a two-sample MR design.*

 - We have added a brief description of what is meant by “summarized data”. 

*A3.    For the description of methods, I would firstly comment that the degree of customisability of these methods (mr_mbe, for example), is rather impressive and to be lauded. However, I would perhaps add one or two sentences to each of the method paragraphs further clarifying the use of these options. For example, the description of the priors of SNPs with causal effects may be quite unclear for those coming new to these methods. While I acknowledge that ultimately the papers behind each method should be read by users of the software, one or two extra helper sentences for each method would be an easy way to improve the ease of use for your software.*

 - We appreciate the sentiment of the reviewer. However, as the reviewer states, detailed descriptions of these methods are already available in the literature, and a paper reviewing the methods has already been written (Slob and Burgess, 2020). Our strong preference is that this paper remains short and focuses on the software package, rather than deviating too strongly into technical detail. To this end, we have included a brief description of the methods in a separate table (Table 2), so that this information is available to the interested reader, but it does not add to the length of the text. The aim of this paper is now more clearly stated in the introduction. 

*A4.    Similarly, it would be useful to add some extra description to the MVMR introduction to explicitly specify what is going on; for example that the basic MVMR is just a regression with first order weights (y ~ x1 +x2) to make it less intimidating for users.*

 - We have added a brief description of multivariable Mendelian randomization (MVMR), and describe how the multivariable IVW method is implemented using multivariable regression. 

*A5.    Is there a facility to report Cochran’s or ruckers Q within the software? The authors discuss the value of visually inspecting plots for heterogeneity but it would wonderful if it were possible to return the statistics for this.*

 - The mr_ivw and mr_egger functions provide the “Heterogeneity test statistic” as part of the outcome. This is the same as Cochrane’s Q statistic for the IVW method and Rücker’s Q statistics for the MR-Egger method. This is clarified in the help files for the mr_ivw and mr_egger functions, and now in the manuscript. 

*A6.    I also note that it is enjoyable that the plots can be directly edited as ggplot objects.*

 - That is wonderful! 

*A7.    Finally, it would be excellent to have a brief paragraph explaining the format of the data frame of results being reported. It can be quite frustrating when using a package to have to manually explore the data object to try and sleuth out what exactly is present and what it means. I expect this is in the vignette in R but I recommend a summary of this here, perhaps, even only for the components reported in common for all MR methods.*

 - We understand the reviewer’s request, and sympathize with this problem. As suggested, we have provided details of some key outputs that are common to all (or at least, most) methods. A full description of the output of each method is readily available in the package documentation.

 - For example, typing ?mr_ivw opens the description of the mr_ivw function. Under the subheading “Value”, there is a clear list of the output for the IVW method. In this case, there are 15 slots: Model, Exposure, Outcome, Correlation, Robust, Penalized, Estimate, StdError, CILower, CIUpper, Alpha, Pvalue, SNPs, RSE, and Heter.Stat. We do not think it would be helpful to the reader to provide a detailed list of every slot for every method in this manuscript, but have added an explanation of how to access this information. 

*A8. In general, this is a welcome update that is described in a clear manner. With the minor updates I suggest above it will be a very useful tool for genetic epidemiology.*

 - We thank the reviewer for his comments, which have strengthened and clarified this manuscript.
